# The Association of Osteosarcopenia With Functional Disability in Community-Dwelling Mexican Adults 50 and Older

**DOI:** 10.3389/fmed.2021.674724

**Published:** 2021-06-07

**Authors:** Miriam T. López-Teros, Oscar Rosas-Carrasco, Sergio Sánchez-García, Lilia Castro-Porras, Armando Luna-López, Marcela Agudelo-Botero

**Affiliations:** ^1^Medical, Dental and Health Sciences Program, National Autonomous University of Mexico, Mexico City, Mexico; ^2^Health Department, Iberoamerican University, Mexico City, Mexico; ^3^Epidemiological Research Unit and Health Services, Aging Area, XXI Century National Medical Center, Mexican Social Security Institute, Mexico City, Mexico; ^4^Politics, Population and Health Research Center, School of Medicine, National Autonomous University of Mexico, Mexico City, Mexico; ^5^Sub-directorate for Biomedical Research, Basic Research Department, National Institute of Geriatrics, Mexico City, Mexico

**Keywords:** sarcopenia, osteosarcopenia, functional disability, community-dwelling, Mexico City

## Abstract

**Background:** Osteosarcopenia (OS) has recently been described as a predictor of negative outcomes in older adults. However, this alteration in body composition has not been widely studied. In Mexico and Latin America, no information is available on its frequency or associated factors.

**Objective:** To analyze the association between OS with FD in community-dwelling Mexican adults 50 and older.

**Design:** Cross-sectional secondary data analysis was performed using primary data from a prospective study Frailty, Dynapenia and Sarcopenia Study in Mexican Adults (FraDySMex).

**Setting and Participants:** Eight hundred and twenty-five people were included, 77.1% women, aged 70.3 ± 10.8 years old.

**Methods:** OS was defined as when the person was diagnosed with sarcopenia (SP) plus osteopenia/osteoporosis. The SP diagnosis was evaluated in accordance with the criteria of the European Working Group for the Definition and Diagnosis of Sarcopenia (EWGSOP), and the osteoporosis diagnosis using World Health Organization (WHO) criteria. Muscle mass and bone mass were evaluated using dual-energy X-ray absorptiometry (DXA). FD was evaluated using the basic activities of daily living (BADL) and the instrumental activities of daily living (IADL). Additional sociodemographic and health co-variables were also included, such as sex, age, education, cognitive status, depression, comorbidity, hospitalization, polypharmacy, urinary incontinence, and nutrition variables such as risk of malnutrition and obesity. Associations between OS with FD were evaluated using multiple logistic regression.

**Results:** The prevalence of OS was 8.9% and that of FD was 8.9%. OS was associated with FD [odds ratio (OR): 1.92; CI 95%: 1.11–3.33].

**Conclusions and Implications:** Comprehensive OS assessment could help clinicians identify risk factors early, and thus mitigate the impact on FD in older people.

## Introduction

Changes in body composition in older people, such as loss of muscle and bone mass, can increase their risk of developing geriatric conditions like sarcopenia (SP) and osteosarcopenia (OS), which is defined as the coexistence of osteopenia/osteoporosis and SP (1, 2). Both OS and SP have been associated with adverse effects on the elderly population, such as frailty, falls, a low quality of life, hospitalization, functional disability (FD), and death (3–7), all of which represent high costs in health systems (3). The muscle and bone tissue loss share several pathophysiological mechanisms which involve a high burden on the health of older adults, leading to the recognition of OS as an emerging geriatric condition (3). It is estimated that, due to the increase in older adults (60 and older) around the world, OS will also increase. The increasing number of falls and fractures will lead to a higher FD in this population (3).

The data around these clinical conditions are heterogeneous and, in some cases in Latin America, data are not available. For SP, the prevalence in Mexico ranges from 9.9 to 33.6% (4–7), while the prevalence of OS has not been reported. However, in other countries OS varies between 5 and 37% among older community-dwelling adults (3). For osteoporosis, the prevalence among Mexican older adults has been reported as, among women and men respectively, between 16 and 6% for osteoporosis of the hip, and 17 and 9% for osteoporosis of the spine (8). Older adults with OS, when compared to older adults who have only SP or osteopenia/osteoporosis, have lower physical performance and an increased risk of fracture, institutionalization, and FD (9–12).

In addition, FD is more common among older people than in the rest of the population (13, 14). A prevalence of FD among Mexican older adults (60 and older) of 26.9% has been reported for the basic activities of daily living (BADL), and 24.6% for the instrumental activities of daily living (IADL) (15). Studies of Mexican older adults have found that risk factors for FD are primarily being an older adult; being female; having polypharmacy, anorexia, weight loss, malnutrition, depression, cognitive impairment, or a comorbidity; a lack of physical activity; and smoking and alcoholism (16–19). No previous study in Mexico has explored the relationship between OS and FD, despite the fact that evidence shows that OS can be a highly predictive geriatric condition in the development of FD in older people (3, 9–12). This is particularly relevant when considering that Mexico is going through an accelerated demographic aging process. In the last 10 years, the proportion of adults 60 years and older increased from 9.1% in 2010 to 12% in 2020, and it is expected that by 2030 one in five people will belong to this age group (20).

The objective of this study is to analyze the association between OS and FD in community-dwelling Mexican adults 50 and older. Few studies have looked at this relationship, but most of those that have, have come from high-income countries. Thus, it is necessary to explore in greater detail the characteristics and epidemiology of OS and FD in a middle-income population such as Mexico, in such a way that prevention and intervention strategies more appropriate to the local context can be developed. Furthermore, while the most unfavorable outcomes due to OS occur in older adults, identifying associated factors earlier can help minimize negative impacts in that group.

## Materials and Methods

### Design and Study Population

This study performs a cross-sectional analysis of women and men 50 years and older, who are community residents and participants in the FraDySMex Study (Frailty, Dynapenia, and Sarcopenia in Mexican Adults). This cohort of community-dwelling adults comes mainly from three municipalities (out of a total of 16) in southeast Mexico City (Cuajimalpa, Magdalena Contreras, and Álvaro Obregón). These three areas hold 12.5% of the total of those 60 and older in Mexico City and have high levels of poverty (Cuajimalpa: 30.1%; Magdalena Contreras: 32.6%; and Álvaro Obregón: 27.9%) (21). Participants were invited to take part in the cohort during home visits made by a psychologist or a social worker, as well as through flyers left in churches, senior community centers, social security centers, and health centers in the designated areas (22).

People eligible to participate in the study were: (1) those who were able to move around with or without assisting devices; and (2) those who were able to answer the study questionnaire by themselves or with the help of a caregiver if their Mini-Mental State Examination (MMSE) score was 10 points or less (23). People were excluded from the study if they were institutionalized, had decreased alertness with any cause, and if they had any acute or chronic condition that, in the judgment of the medical staff, could affect their ability to answer the proposed questionnaire and complete the objective evaluation. Also, people without grip strength tests or a dual-energy X-ray absorptiometry (DXA) body composition assessment were also excluded.

**Figure 1:**
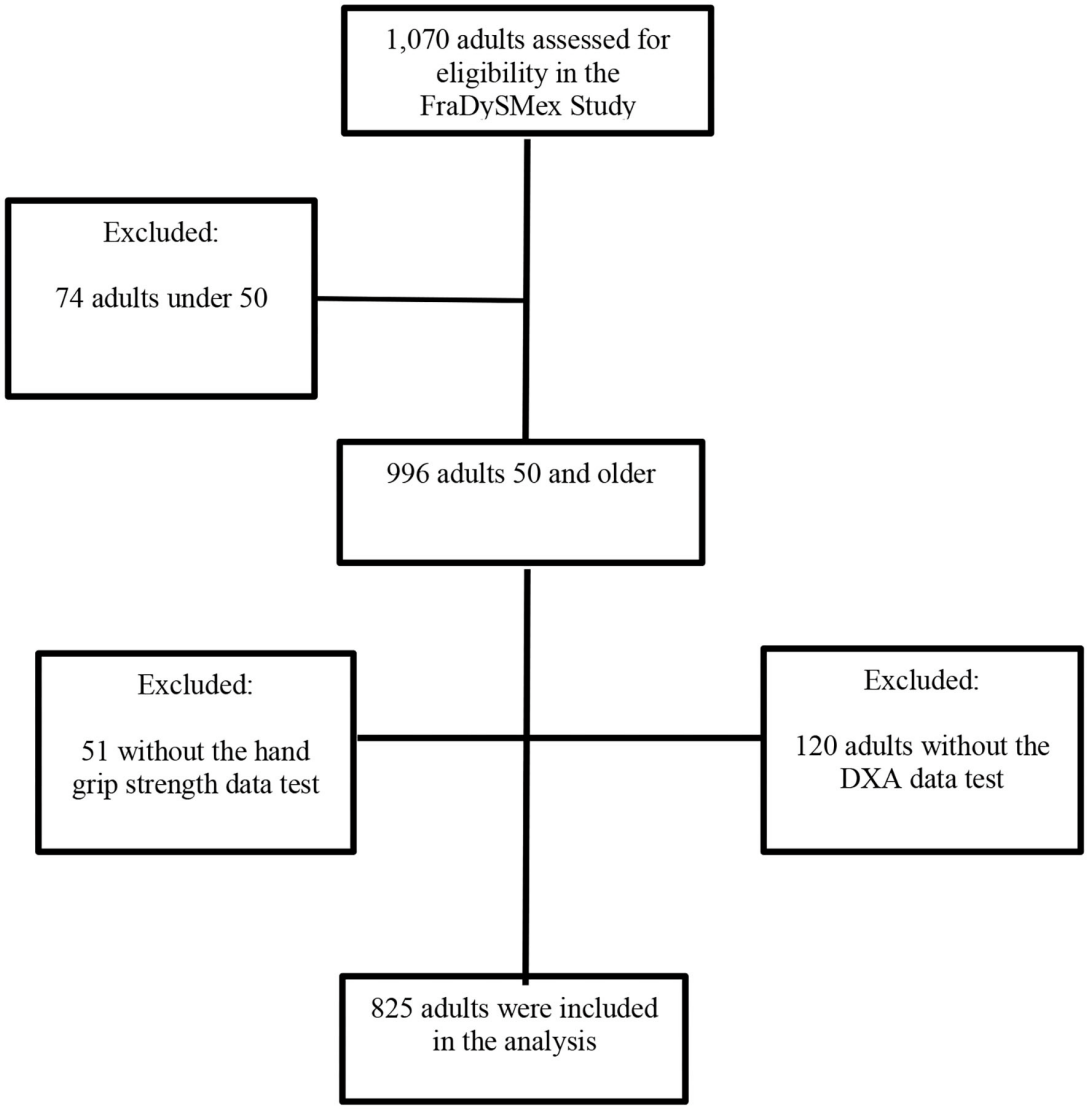
Flowchart of FraDySMex (Frailty, Dynapenia, and Sarcopenia in Mexican adults Study (FraDySMex). DXA, dual-energy X-ray absorptiometry.

The study had a 3-round design. The first round consisted of the assessment of individuals from October 2014 to December 2015 (*n* = 606), and the second round from October to December 2019 (*n* = 1,070). In this last round, new people were added to the cohort and some individuals who had participated in the first round were reevaluated. The participants were received in the Research Laboratory on Functional Evaluation at the National Institute of Geriatrics and the Older Adult Evaluation Center at the Iberoamerican University in Mexico City. There, the medical staff, composed of geriatricians, internists, general practitioners, nurses, physical therapists, nutritionists, and specialists in geriatric rehabilitation, conducted a series of objective evaluations on participants. The selection of the study population is shown in Figure 1.

### Measurements

#### Functional Disability Assessment

FD was evaluated using the Barthel scale for BADL and the Lawton scale for IADL (24, 25). A score of ≤90 on the BADL scale or ≥1 on the IADL scale were considered as FD.

#### Osteosarcopenia Definition

OS is a condition that describes the co-existence of osteoporosis and sarcopenia, two chronic musculoskeletal conditions associated with aging (2, 3). The sarcopenia was defined according to the criteria of the European Working Group on Sarcopenia in Older People (EWGSOP) (1), adjusted to the population studied, considering low strength, muscle mass and poor physical performance (slow gait speed) (1). Total skeletal muscle mass (kg) (SMT) and appendicular skeletal muscle mass (arms and legs) (kg) (ASM) was measured using dual-energy X-ray absorptiometry (DXA) (Hologic Discovery-WI; Hologic Inc, Bedford-MA). A manual dynamometer (JAMAR Hydraulic Hand Dynamometer, Lafayette, IN) was used to measure manual pressure force; three measurements were taken from each arm and the highest was considered for the analysis. The gait speed was measured for physical performance, which was recorded from a regular six-meter pace walk on the GAIT Rite instrumented mat (platinum 20) (204 × 35.5 × 0.25 inches, 100 Hz sample rate). The cut-off points used for these variables are described in Table 1. To determine osteopenia/osteoporosis, DXA was used to measure the total bone mineral density (g/cm^2^) (BMD), the femur, and the lumbar spine (L1–L5). WHO criteria were used to diagnose osteopenia/osteoporosis, osteoporosis was considered when the T-score was lower than 2.5 SDs (standard deviations) and osteopenia when the T-score was between 2.5 and 1.0 SDs of the lumbar spine or femur BMD below that of the reference population of young adults (26).

**Table 1:** Components and cut-off points used for the diagnosis of sarcopenia.

**Sex**	**ASM^a^**	**Gait speed^b^**	**Hand-grip strength^c^**	
Males	ASM ≤ 6.68 kg/m^2^	Height ≤ 1.65 m ≥ 5.7 s	BMI ≤ 24.3kg/m^2^	≤ 22
		Height > 1.65 m ≥ 4.5 s	BMI 24.4–26.6kg/m^2^	≤ 22
			BMI 26.7–28.5 kg/m^2^	≤ 24
			BMI > 28.5 kg/m^2^	≤ 22
Females	ASM ≤ 5.35 kg/m^2^	Height ≤ 1.51 m ≥ 6.8 s	BMI ≤ 24.7 kg/m^2^	≤ 12
		Height > 1.51 m ≥ 5.4 s	BMI 24.8–27.6 kg/m^2^	≤ 12
			BMI 27.7–30.5 kg/m^2^	≤ 12
			BMI > 30.5 kg/m^2^	≤ 13

*ASM, Appendicular skeletal muscle mass; BMI, Body mass index*.

a *Cut-off points according to the lowest quintile of ASM*.

b *Cut-off points by height according to the lowest quintile of gait speed*.

c *Cut-off points by BMI quartile*.

### Co-variates

#### Sociodemographic

Age (years), sex, and schooling (<10; ≥10 years).

#### Health Conditions

Depressive symptoms, from the Depression Scale of the Center for Epidemiological Studies (CESD-7 scale); depression was considered if subjects scored five or more (27). Cognitive status, which was assessed using the MMSE (cognitive impairment was considered when ≤23 points were obtained with 5 years of school education, ≤19 points with between 1 and 4 years of education, ≤16 without education or with <1 year of education) (28). Comorbidity was assessed using the Charlson Comorbidity Index, adapted to Mexican Spanish (≥3 points was considered high comorbidity) (29, 30). Polypharmacy was defined as taking five or more medications (31), and urinary incontinence was defined using the incontinence items on the Barthel scale (24).

#### Nutrition Variables and Body Composition

Malnutrition was assessed through the Mini Nutritional Assessment (MNA) test, using a cut-off point of ≤23 (risk of malnutrition) (32). The percentage of total body fat was used for women ≥40% and men ≥30% for obesity measured by DXA (33). In addition, anthropometric measurements such as weight, size and BMI (body mass index) were also used to adjust muscle strength and gait components of the sarcopenia diagnosis.

#### Physical Activity

Low physical activity was defined using the lowest quintile of kilocalories per week, obtained via the physical activity questionnaire for older adults (CHAMPS); <545.7 for men and <481.2 kcal/week for women (34).

### Statistical Analysis

Variables were described by arithmetic means and standard deviation (SD) or proportions as appropriate. Group differences between participants with or without FD were evaluated using the *t-Student* test or the *Chi-squared* test for continuous and categorical variables. Logistic regression models (adjusted and not adjusted) were used to determine the association between SP and OS with FD, and the results are shown in terms of an odds ratio (OR). We included the known factors that may modify the effect of this association and that have been previously described in the literature. The variables included in the final models were those significantly related with FD in bivariate analysis. The model with the best fit was chosen. Differences were considered statistically significant with *p* ≤ 0.05, and confidence intervals (CI) were also reported at 95%. Likewise, collinearity and interaction between variables were also verified for the final models. The data was analyzed using *Stata version 18*^®^ (Stata Corp, College Station, Texas, USA).

## Results

The average age of the participants was 70.3 ± 10.8 years; 77.1% were women and 52.2% had <10 years of schooling. Regarding the health characteristics of the study population, the following incidences were found: cognitive impairment (10.9%), depression (28.8%), high comorbidity (22.3%), polypharmacy (33.1%), and urinary incontinence (8.9%). In terms of nutrition variables, 30.3% of participants were at risk of malnutrition and 53.5% of obesity. Of the total sample, 8.9% had FD. In addition, the prevalence of SP and OS was 14.9 and 8.9%, respectively. In the comparative analysis between groups (with and without FD), the variables that were significant were: age, sex (women), low education, cognitive impairment, depression, polypharmacy, high comorbidity, urinary incontinence, hospitalization, risk of malnutrition, low physical activity, SP, and OS (Table 2).

**Table 2:** Characteristics of participants by functional disability.

**Characteristics**	**Total**	**With functional disability**		**Without functional disability**		***p*-value**
	***N* [95% CI]**	***N***	**% [95% CI]**	***N***	**% [95% CI]**	
Sarcopenia	15 [12–17]	36	19 [13–24]	69	10 [7–13]	0.00
Osteosarcopenia	9 [7–11]	29	17 [11–23]	45	6 [4–8]	0.00
**Sociodemographic**						
Age, years	69.9 ± 9.3	207	77.5 ± 9.5	707	67.1 ± 9.4	0.00
50–65 years old	52 [46–53]	46	61 [54–68]	430	22 [16–29]	
>65 years old	48 [42–49]	161	78 [72–83]	277	39 [36–43]	0.00
Women	78 [75–80]	166	80 [74–81]	556	78 [75–81]	0.68
Low education < 10 years	58 [49–56]	146	70 [64–77]	331	46 [43–50]	0.00
**Health conditions**						
Cognitive impairment (MMSE)^*^	11 [9–13]	45	24 [18–31]	47	7 [5–9]	0.00
Depression (CESD-7 ≥ 5)	29 [26–32]	75	41 [34–48]	168	25 [21–28]	0.00
High Comorbidity (Charlson Comorbidity Index ≥ 3 points)	23 [19–25]	66	34 [29–43]	123	18 [15–21]	0.00
Hospitalization, ≥1 in the last year	12 [9–13]	41	19 [14–25]	62	8 [6–10]	0.00
Polypharmacy, ≥5 medications	31 [30–36]	92	50 [43–58]	189	28 [24–31]	0.00
Urinary incontinence	10 [7–11]	47	22 [16–28]	34	4 [3–6]	0.00
**Other nutrition and body composition variables**						
Risk of malnutrition (MNA ≤ 23)	30 [27–33]	86	50 [42–57]	163	24 [21–28]	0.00
Obesity (≥40% women and ≥35% men)	59 [54–60]	122	58 [52–65]	367	51 [48–55]	0.07
Low physical activity	20 [18–23]	64	3 [25–37]	60	8 [2–15]	0.00

*CI, Confidence intervals*.

* *MMSE, Mini-Mental State Examination (cognitive impairment was considered when ≤23 points were obtained with 5 years of school education, ≤19 points with between 1 and 4 years of schooling, ≤16 without schooling or with <1 year of schooling)*.

*CESD, Depression Scale of the Center for Epidemiological Studies*.

*MNA, Mini Nutritional Assessment*.

**Figure 2:**
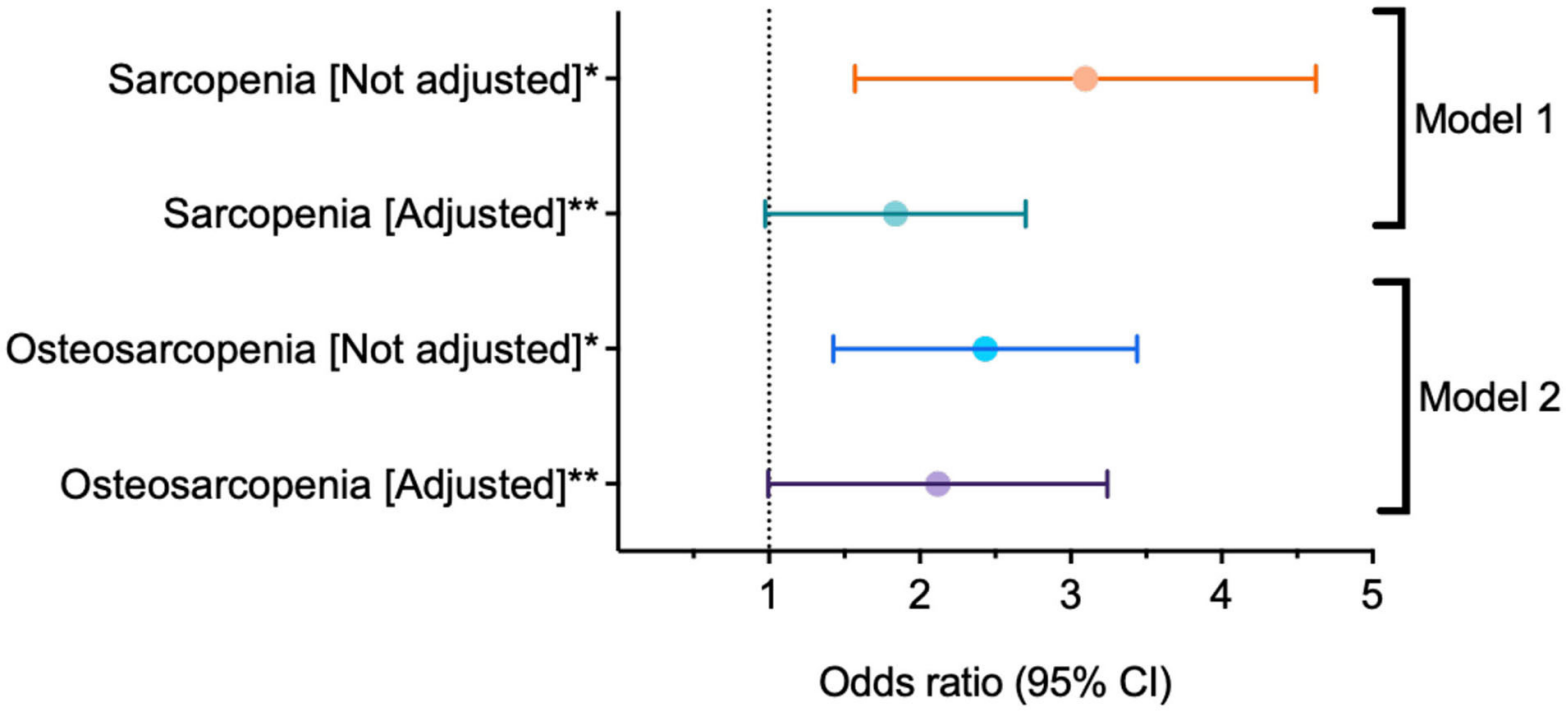
Logistic regression between sarcopenia, osteosarcopenia, and functional disability. CI, Confidence intervals. *p < 0.001; **p < 0.05. Both models were adjusted by age, sex, polypharmacy, risk of malnutrition, and low physical activity.

In the multivariate analysis, an increased risk of FD was found in adults with SP (OR: 1.70, CI 95%: 1.03–2.81, *p* = 0.04), an association that was higher in adults with OS (OR: 1.94, CI 95%: 1.10–3.42, *p* = 0.02), after adjusting for age, sex, polypharmacy, risk of malnutrition, and low physical activity (Figure 2).

## Discussion

The objective of this study was to analyze the association between OS and FD in community-dwelling adults 50 years and older in Mexico City. Our results suggest that there is a statistically significant association between OS and FD (after adjusting for age, polypharmacy, and risk of malnutrition; known factors that may modify the effect of disability association), and that this association is higher than that in those adults with SP only. This coincides with the results of other studies. For example, Kirk et al. (3) found that Australian older people with OS were 2.6 times more at risk of developing FD than those without OS; however, that study doesn't compare that risk vs. SP alone. Drey et al. (35) showed that in pre-frail older adults, osteosarcopenic individuals had a significant reduction in physical performance, suggesting that adults with OS have a higher risk for further functional decline when compared to sarcopenic and osteopenic/osteoporotic individuals. It is important to reiterate that there are few studies published to date that analyze the relationship between OS and FD. Furthermore, our results corroborate the conclusion that OS represents a greater risk for FD in our study's population than SP alone.

During the aging process, there are several changes in body composition, both on a molecular and tissue level. This is either due to an increase and redistribution of adipose tissue or to a loss of fat free body mass (particularly at the expense of muscle and bone mass). These changes determine the risk of developing geriatric syndromes such as SP and OS; public health problems that impact the quality of life in the geriatric population (1–4). It has also been reported that these syndromes have common risk factors and outcomes and generally interact with each other as OS; that is, one syndrome can contribute to the onset of another and incur worse results, such as functional repercussions and a loss of independence in older adults (3, 12, 35).

This is the first study in Mexico to analyze the relationship between OS with FD in older adults, although previously an association of osteosarcopenic obesity (OSO) syndrome with low physical performance and frailty had been shown as risk factors for FD among older Mexican adult women (36). This evidence indicates the importance of integrating a body composition measure, as a risk factor for FD, into the geriatric assessment. Timely detection of changes in muscle and bone tissue in older adults can help health personnel start a specific treatment (diet, physical activity, and promoting healthy behaviors) that prevents further deterioration, development of SP and OS, and subsequently FD.

It should be noted that in Mexico, this assessment of body composition (measurement of muscle mass, fat mass and bone mass) is not included in most primary and secondary care levels. Not all centers have the necessary infrastructure to perform these measurements, such as electrical bioimpedance equipment or dual-energy X-ray absorptiometry. Also, few health personnel are trained to perform and interpret these measurements in the population of older adults. Therefore, it is necessary to develop and implement protocols or algorithms based on the evaluation of body composition indicators as early predictors of FD in older adults. Similarly, it is essential to generate *ad hoc* scales or measuring systems for the Mexican population that can be used by medical personnel in health institutions where specialized equipment such as those used in this research is not available.

FD has a higher prevalence and impact on the older adult population; however, it is necessary to detect risk factors at younger ages, such as loss of muscle and bone mass starting at around age 40 or 50 and increasing with advancing age. These physiological and molecular changes begin gradually, such as with the alteration of hormones and inflammation factors, which is accentuated after the age of 60 (33). Detection of these changes at younger ages allows for effective treatments to be implemented to prevent the development of geriatric conditions like FD.

We consider it necessary to conduct further studies on this topic and to include other phenotypes associated with body composition in the analysis, such as sarcopenia obesity, OSO, and other FD-related sociodemographic variables such as socioeconomic level, race, ethnic group, quality of life and access to health services. Likewise, it is recommended to conduct this analysis with a longitudinal design and with different types of geriatric populations.

This study has some limitations that should be considered. First, the analysis used was cross-sectional; therefore, no conclusions on causality between FD and OS could be reached. However, one strength is that 825 participants were included, which could establish a significant association between the variables of interest. A second limitation is that FD was measured through self-reported scales based on limitations in BADL and IADL. A final limitation has to do with a possible selection bias, since the sample consisted of adults who were able to go, on their own, to the centers where the evaluations were carried out, and those adults who were less healthy and with a higher degree of FD could have been excluded.

## Conclusion and Implications

Comprehensive OS assessment could help clinicians identify risk factors early and thus mitigate the impact on FD in older people. FD is one of the most relevant health indicators of older adults, not only due to individual impacts, but also because of the increase in dependency and the costs implied for health systems, especially in middle- and lower-income countries. OS is a geriatric syndrome that, together with other syndromes, should be evaluated to design more appropriate interventions, based on the specific needs of the older Mexican population.

## Data Availability Statement

The raw data supporting the conclusions of this article will be made available by the authors, without undue reservation.

## Ethics Statement

This study was approved by the Ethics Committee of the Angeles Mocel General Hospital, and registered by the National Institute of Geriatrics (DI-PI-002/2014), as well as with the National Bioethics Commission (CONBIOETICA-09-cei-013-20170517/2019). The informed written consent of all individuals was obtained.

## Author Contributions

ML-T contributed to the data collection, statistical data analysis, and manuscript writing. OR-C was responsible for the study design (FraDySMex) project, study approval in the ethics committee, data collection, and manuscript review. SS-G contributed data analysis and manuscript review. LC-P contributed to statistical analysis. AL-L contributed to the final manuscript review. MA-B is the corresponding author and contributed to writing and reviewing the manuscript. All authors contributed to the article and approved the submitted version.

## Conflict of Interest

The authors declare that the research was conducted in the absence of any commercial or financial relationships that could be construed as a potential conflict of interest.

## References

[1] Cruz-Jentoft AJ Bahat G Bauer J Boirie Y Bruyère O Cederholm T. Sarcopenia: revised European consensus on definition and diagnosis. Age Ageing. (2019) 48:16–31. 10.1093/ageing/afy169 30312372 PMC6322506

[2] Paintin J Cooper C Dennison E. Osteosarcopenia. Br J Hosp Med. (2018) 79:253–8. 10.12968/hmed.2018.79.5.253 PMC5963675 29727228

[3] Kirk B Zanker J Duque G. Osteosarcopenia: epidemiology, diagnosis, and treatment-facts and numbers. J Cachexia Sarcopenia Muscle. (2020) 11:609–18. 10.1002/jcsm.12567 32202056 PMC7296259

[4] Manrique-Espinoza B Salinas-Rodríguez A Rosas-Carrasco O Gutiérrez-Robledo LM Avila-Funes JA. Sarcopenia is associated with physical and mental components of health-related quality of life in older adults. J Am Med Dir Assoc. (2017) 18:636.e1–e5. 10.1016/j.jamda.2017.04.005 28549718

[5] Velázquez Alva MC Irigoyen Camacho ME Lazarevich I Delgadillo Velasquez J Acosta Dominguez P Zepeda Zepeda MA. Comparison of the prevalence of sarcopenia using skeletal muscle mass index and calf circumference applying the European consensus definition in elderly Mexican women. Geriatr Gerontol Int. (2017) 17:161–70. 10.1111/ggi.12652 26534889

[6] Parra-Rodríguez L Szlejf C García-González AI Malmstrom TK Cruz-Arenas E Rosas-Carrasco O. Cross-cultural adaptation and validation of the spanish-language version of the SARC-F to assess sarcopenia in Mexican Community-Dwelling Older Adults. J Am Med Dir Assoc. (2016) 17:1142–6. 10.1016/j.jamda.2016.09.008 27815111

[7] Espinel-Bermúdez MC Sánchez-García S García-Peña C Trujillo X Huerta-Viera M Granados-García V. Factores asociados a sarcopenia en adultos mayores mexicanos: encuesta Nacional de Salud y Nutrición (2012). Rev Med Inst Mex Seguro Soc. (2018) 56(Suppl. 1):46–53. 10.1590/S0036-36342011000100004 29624960

[8] Clark P Tamayo JA Cisneros F Rivera FC Valdés. Epidemiology of osteoporosis in Mexico. Present and future directions. Rev Invest Clin. (2013) 65:183–91. 23844537

[9] Kilavuz A Meseri R Savas S Simsek H Sahin S Bicakli DH. Association of sarcopenia with depressive symptoms and functional status among ambulatory community-dwelling elderly. Arch Gerontol Geriatr. (2018) 76:196–201. 10.1016/j.archger.2018.03.003 29550658

[10] Tanimoto Y Watanabe M Sun W Sugiura Y Hayashida I Kusabiraki T. Sarcopenia and falls in community-dwelling elderly subjects in Japan: defining sarcopenia according to criteria of the European Working Group on Sarcopenia in Older People. Arch Gerontol Geriatr. (2014) 59:295–9. 10.1016/j.archger.2014.04.016 24852668

[11] Tyrovolas S Koyanagi A Olaya B Ayuso-Mateos JL Miret M Chatterji S. Factors associated with skeletal muscle mass, sarcopenia, and sarcopenic obesity in older adults: a multi-continent study. J Cachexia Sarcopenia Muscle. (2016) 7:312–21. 10.1002/jcsm.12076 27239412 PMC4864288

[12] Huo YR Suriyaarachchi P Gomez F Curcio CL Boersma D Muir SW. Phenotype of osteosarcopenia in older individuals with a history of falling. J Am Med Dir Assoc. (2015) 16:290–5. 10.1016/j.jamda.2014.10.018 25512216

[13] World Health Organization. Informe Mundial Sobre la Discapacidad. (2014). Available online at: https://www.who.int/disabilities/world_report/2011/es/

[14] Martinez-Gomez D Guallar-Castillon P Rodríguez-Artalejo F. Sitting time and mortality in older adults with disability: a National Cohort Study. J Am Med Dir Assoc. (2016) 17:960.e15–20. 10.1016/j.jamda.2016.07.016 27592178

[15] Manrique-Espinoza B Salinas-Rodríguez A Moreno-Tamayo KM Acosta-Castillo I Sosa-Ortiz AL Gutiérrez-Robledo LM. Condiciones de salud y estado funcional de los adultos mayores en México [Health conditions and functional status of older adults in Mexico]. Salud Publica Mex. (2013) 55(Suppl. 2):S323–31. 10.21149/spm.v55s2.5131 24626711

[16] Menéndez J Guevara A Arcia N León Díaz EM Marín C Alfonso JC. Enfermedades crónicas y limitación funcional en adultos mayores: estudio comparativo en siete ciudades de América Latina y el Caribe [Chronic diseases and functional limitation in older adults: a comparative study in seven cities in Latin America and the Caribbean]. Rev Panam Salud Publica. (2005) 17:353–61. 10.1590/S1020-49892005000500007 (in Spanish). 16053645

[17] Murphy RA Reinders I Register TC Ayonayon HN Newman AB Satterfield S. Associations of BMI and adipose tissue area and density with incident mobility limitation and poor performance in older adults. Am J Clin Nutr. (2014) 99:1059–65. 10.3945/ajcn.113.080796 24522448 PMC3985211

[18] Okabe T Abe Y Tomita Y Mizukami S Kanagae M Arima K. Age-specific risk factors for incident disability in activities of daily living among middle-aged and elderly community-dwelling Japanese women during an 8-9-year follow up: the Hizen-Oshima study. Geriatr Gerontol Int. (2017) 17:1096–101. 10.1111/ggi.12834 27401720

[19] Wu LW Chen WL Peng TC Chiang ST Yang HF Sun YS. All-cause mortality risk in elderly individuals with disabilities: a retrospective observational study. BMJ Open. (2016) 6:e011164. 10.1136/bmjopen-2016-011164 27625055 PMC5030612

[20] Censo de Población y Vivienda (2020). Available online at: https://www.inegi.org.mx/programas/ccpv/2020/

[21] Coneval: Consejo Nacional de Evaluación de la Política de Desarrollo Social. Medición de la Pobreza (2015). Retrieved from: https://www.coneval.org.mx/Medicion/Paginas/PobrezaInicio.aspx

[22] Rosas-Carrasco O Cruz-Arenas E Parra-Rodríguez L García-González AI Contreras-González LH Szlejf C. Cross-cultural adaptation and validation of the FRAIL Scale to assess frailty in Mexican adults. J Am Med Dir Assoc. (2016) 17:1094–8. 10.1016/j.jamda.2016.07.008 27567463

[23] Ostrosky-Solís F López-Arango G Ardila A. Sensitivity and specificity of the Mini-Mental State Examination in a Spanish-speaking population. Appl Neuropsychol. (2000) 7:25–31. 10.1207/S15324826AN0701_4 10800625

[24] Mahoney FI Barthel DW. Functional evaluation: the Barthel Index. Md State Med J. (1965) 14:2. 10.1037/t02366-000 14258950

[25] Lawton MP Brody EM. Assessment of older people: self-maintaining and instrumental activities of daily living. Gerontologist. (1969) 9:179–86. 10.1093/geront/9.3_Part_1.179 5349366

[26] World Health Organization. Assessment of fracture risk and its application to screening for postmenopausal osteoporosis. Report of a WHO Study Group. World Health Organ Tech Rep Ser. (1994) 843:1–129. 7941614

[27] Salinas-Rodríguez A Manrique-Espinoza B Acosta-Castillo GI Franco-Nuñez A Rosas-Carrasco O Gutiérrez-Robledo LM. Validation of a cutoff point for the short version of the Depression Scale of the Center for Epidemiologic Studies in older Mexican adults. Salud Publica Mex. (2014) 56:279–85. 10.21149/spm.v56i3.7346 25272180

[28] Folstein MF Folstein SE McHugh PR. “Mini-mental state”. A practical method for grading the cognitive state of patients for the clinician. J Psychiatr Res. (1975) 12:189–98. 10.1037/t07757-000 1202204

[29] Charlson ME Pompei P Ales KL MacKenzie CR. A new method of classifying prognostic comorbidity in longitudinal studies: development and validation. J Chronic Dis. (1987) 40:373–83. 10.1016/0021-9681(87)90171-8 3558716

[30] Rosas-Carrasco O González-Flores E Brito-Carrera AM Vázquez-Valdez OE Peschard-Sáenz E Gutiérrez-Robledo LM. Assessment of comorbidity in the elderly. Rev Med Inst Mex Seguro Soc. (2011) 49:153–62. 21703142

[31] Shah BM Hajjar ER. Polypharmacy, adverse drug reactions, and geriatric syndromes. Clin Geriatr Med. (2012) 28:173–86. 10.1016/j.cger.2012.01.002 22500537

[32] Cuyac-Lantigua M Santana-Porbén S. The Mini Nutritional Assessment of the elderly in the practice of a hospital geriatrics service: inception, validation and operational characteristics. Arch Latinoam Nutr. (2007) 57:255–65. 18271404

[33] Dufour AB Hannan MT Murabito JM Kiel DP McLean RR. Sarcopenia definitions considering body size and fat mass are associated with mobility limitations: the Framingham Study. J Gerontol A Biol Sci Med Sci. (2013) 68:168–74. 10.1093/gerona/gls109 22503991 PMC3598358

[34] Stewart AL Mills KM King AC Haskell WL Gillis D Ritter PL. CHAMPS physical activity questionnaire for older adults: outcomes for interventions. Med Sci Sports Exerc. (2001) 33:1126–41. 10.1097/00005768-200107000-00010 11445760

[35] Drey M Sieber CC Bertsch T Bauer JM Schmidmaier R FiAT intervention group. Osteosarcopenia is more than sarcopenia and osteopenia alone. Aging Clin Exp Res. (2016) 28:895–9. 10.1007/s40520-015-0494-1 26563287

[36] Szlejf C Parra-Rodríguez L Rosas-Carrasco O. Osteosarcopenic obesity: prevalence and relation with frailty and physical performance in middle-aged and older women. J Am Med Dir Assoc. (2017) 18:733.e1–e5. 10.1016/j.jamda.2017.02.023 28431912

